# “Combo” Multi-Target Pharmacological Therapy and New Formulations to Reduce Inflammation and Improve Endogenous Remyelination in Traumatic Spinal Cord Injury

**DOI:** 10.3390/cells12091331

**Published:** 2023-05-06

**Authors:** Marzia Moretti, Riccardo Caraffi, Luca Lorenzini, Ilaria Ottonelli, Michele Sannia, Giuseppe Alastra, Vito Antonio Baldassarro, Alessandro Giuliani, Jason Thomas Duskey, Maura Cescatti, Barbara Ruozi, Luigi Aloe, Maria Angela Vandelli, Luciana Giardino, Giovanni Tosi, Laura Calzà

**Affiliations:** 1 Department of Veterinary Medical Science (DIMEVET), University of Bologna, Ozzano Emilia, 40064 Bologna, Italy; marzia.moretti3@unibo.it (M.M.); luca.lorenzini8@unibo.it (L.L.); giuseppe.alastra2@unibo.it (G.A.); vito.baldassarro2@unibo.it (V.A.B.); a.giuliani@unibo.it (A.G.); luciana.giardino@unibo.it (L.G.); 2 Clinical and Experimental Medicine PhD Program, University of Modena and Reggio Emilia, 41125 Modena, Italy; riccardo.caraffi@unimore.it; 3 Nanotech Lab, Te.Far.T.I., Department of Life Sciences, University of Modena and Reggio Emilia, 41125 Modena, Italy; ilaria.ottonelli@unimore.it (I.O.); jasonthomas.duskey@unimore.it (J.T.D.); ruozi.barbara@unimore.it (B.R.); mariaangela.vandelli@unimore.it (M.A.V.); 4 IRET Foundation, Ozzano Emilia, 40064 Bologna, Italy; michele.sannia@iret-foundation.org (M.S.); maura.cescatti@iret-foundation.org (M.C.); luigi.aloe@cnr.it (L.A.); 5 Health Science and Technologies, Interdepartmental Center for Industrial Research (HST-ICIR), University of Bologna, Ozzano Emilia, 40064 Bologna, Italy; 6 Montecatone Rehabilitation Institute, 40026 Imola, Italy; 7 Department of Pharmacy and Biotechnology (FaBit), University of Bologna, 40126 Bologna, Italy

**Keywords:** spinal cord injury, remyelination, nanomedicines, thyroid hormone, ibuprofen, NGF

## Abstract

Spinal cord injury (SCI) is characterized by a cascade of events that lead to sensory and motor disabilities. To date, this condition is irreversible, and no cure exists. To improve myelin repair and limit secondary degeneration, we developed a multitherapy based on nanomedicines (NMeds) loaded with the promyelinating agent triiodothyronine (T3), used in combination with systemic ibuprofen and mouse nerve growth factor (mNGF). Poly-L-lactic-co-glycolic acid (PLGA) NMeds were optimized and loaded with T3 to promote sustained release. In vitro experiments confirmed the efficacy of T3-NMeds to differentiate oligodendrocyte precursor cells. In vivo rat experiments were performed in contusion SCI to explore the NMed biodistribution and efficacy of combo drugs at short- and long-term post-lesion. A strong anti-inflammatory effect was observed in the short term with a reduction of type M1 microglia and glutamate levels, but with a subsequent increase of TREM2. In the long term, an improvement of myelination in NG2-IR, an increase in MBP content, and a reduction of the demyelination area were observed. These data demonstrated that NMeds can successfully be used to obtain more controlled local drug delivery and that this multiple treatment could be effective in improving the outcome of SCIs.

## 1. Introduction

Spinal cord injury (SCI) occurs when trauma or a disease state damages the spinal cord, often resulting in partial or complete paralysis of the limbs and having a drastic impact on the individual’s quality of life. Medical, psychological, social, and economic burdens on the patients and their families are enormous due to the residual motor, sensory, and cognitive handicaps that occur. The World Health Organization (WHO) reported that the global incidence of SCIs is between 250,000 and 500,000 per year, of which only 35% eventually return to active employment (https://www.who.int/news-room/fact-sheets/detail/spinal-cord-injury; accessed on 2 March 2023), and only 0.7% of patients experience complete neurological recovery. In the EU, the incidence rate is about 10,000 newly injured people per year, with more than 200,000 patients living with SCI (NISCI, 2020), resulting in a very high economic burden [1].

SCI symptoms evolve according to different pathogenetic mechanisms over a specific timeline. The primary phase is characterized by ischemic damage, reflecting the trauma suffered, which develops within minutes and continues for 8–12 h. This phase is characterized by the increase of extracellular excitatory amino acid levels and the tissue infiltration of neutrophils and macrophages. This promotes self-propagating events that determine the secondary phase, or secondary injury. Mechanistically, neuroinflammation, excitotoxicity, mitochondrial dysfunction, oxidative stress, lipid peroxidation, demyelination, axon degeneration, and apoptotic cell death are followed by scar formation, neurodegeneration, and central cavitation, composing the secondary injury that leads to lesion progression outside the trauma area and finally determining the functional outcome [2]. This well-established phenomenon in experimental SCI has now been recognized and described in humans by MRI analysis, including signs of demyelination propagation rostrally to the lesion site [3]. SCI, such as traumatic injuries of the brain, are still unmet clinical needs since no cure, disease-modifying treatments, or regenerative therapies exist, and SCI is in fact listed as “orphan disorders” in the orphaned portal (ORPHA:90058). 

Interrupting or mitigating secondary degeneration has been identified as a primary goal to limit disabilities after central nervous system (CNS) lesions [4,5]. For example, the use of steroids as anti-inflammatory drugs has been extensively proposed for clinical use; however, because of the small benefit/risk ratio and the gastrointestinal side effects, steroids have been abandoned and considered a “harmful standard of care” in many trauma centers [6,7]. 

Remyelination is another major target in SCI pharmacological therapy [8,9]. In fact, demyelination impacts axon integrity and function, leading to retrograde neuronal degeneration and the remote, widespread atrophy of brain gray matter [10]. In experimental SCIs, a rearrangement of gene transcription encoding for synaptic proteins has been described in CNS areas outside and far from the lesion, such as the cerebral cortex, basal ganglia, and cerebellum [11]. Accordingly, remyelination also became a target for neuroprotection [12]. 

Our lab extensively studied the use of thyroid hormones (THs) as a promyelinating agent in preclinical rodents and non-human primate models of inflammatory-demyelinating diseases. On the basis of these results [13,14,15], numerous independent labs have demonstrated that TH supplementation (by triiodothyronine (T3), thyroxine (T4), or TH analogs) favors myelin protection/repair, overcoming the oligodendrocyte precursor cell (OPC) differentiation block, protecting myelin and axons, and ameliorating the clinical outcome in experimental models of inflammatory/demyelinating diseases [15]. These preclinical results led to a phase 1 study for the use of liothyronine in multiple sclerosis (NCT02760056) [16]. However, the risk of hyperthyroidism and thyroid function suppression hampers systemic TH administration as a promyelination strategy.

Therefore, we developed a strategy to interfere with more than one of the pathogenic mechanisms that occur in the early phase of the lesion, with the purpose of limiting secondary degeneration and improving the structural and functional outcome. Moreover, we also developed drug delivery systems (DDSs) based on nanomedicines in order to reduce side effects related to long-term systemic administration of multiple drugs. Nanomedicines (NMeds) are DDSs in the nanoscale range that can be employed to deliver therapeutics to specific targets in a controlled manner and are nowadays one of the most investigated DDSs for the sustained release of active molecules [17,18,19]. Other advantages of NMeds lie in the possibility of having a controllable size and size distribution [20], drug loading capacity [21], controlled drug release [22], and cell-targeting ability [23]. In this proof-of-concept study, we focused on improving myelin repair as the main target of the interventions. Thus, we developed polymer-based NMeds loaded with T3 that were administered as a single dose in the subdural space to obtain a local, prolonged release of T3. In the 3× “combo” therapy, we included systemically administered Ibuprofen (Ibu), a non-steroid anti-inflammatory drug (NSAID), which has been included in the Phase I SCISSOR clinical trial (NCT02096913) [24,25], and the mouse nerve growth factor (mNGF) via subdural and intravenous administration. This 3× “combo” therapy was effective in reducing secondary degeneration triggering mechanisms, reducing glutamate tissue levels and microglia type 1 activation, and improving long-term locomotor functional outcomes by favoring myelin protection/repair.

## 2. Materials and Methods

### 2.1. Preparation and Characterization of NMeds

#### 2.1.1. Preparation of Empty NMeds (Vehicle Control)

Empty NMeds, composed of poly-lactic-co-glycolic acid (PLGA), were obtained in accordance with the nanoprecipitation procedure. Briefly, 50 mg of PLGA (Evonik Industries AG, Essen, Germany) were dissolved in 4 mL of a mixture of acetone and dimethyl sulfoxide (3:1 *v*/*v*) (Merck Life Science, Milano, Italy). The obtained organic phase was added dropwise to an aqueous solution (12.5 mL) of Pluronic^®^ F68 1.5% *w*/*v* (Merck Life Science, Milano, Italy) and kept under stirring (750 rpm) at room temperature. After 10 min of stirring at room temperature, the organic solvent was completely evaporated under reduced pressure (−76 mmHg) by using a rotary evaporator (Buchi Rotavapor R-114, Buchi Vacuum Pump V-300, Buchi Italia, Milano, Italy). PLGA NMeds were recovered and purified by centrifugation (ALC multispeed centrifuge PK 121, Opto-Lab, Modena, Italy) carried out at 9700 rpm for 10 min at room temperature to remove the unformulated materials. The purified NMeds were resuspended in water (4 mL) and stored at −20 °C with the addition of trehalose dihydrate (Merck Life Science) as cryoprotectant (NMeds:trehalose 1:1 *w*/*w*) until further use.

#### 2.1.2. Preparation of T3-NMeds

T3-loaded NMeds were prepared and purified using the same procedures described for empty NMeds (see Section 2.1.1) with the addition of 5 mg of T3 (Fisher Scientific Italia, Milano, Italy) to the organic phase.

#### 2.1.3. Preparation of Cy5-Labeled NMeds

Cy5-labeled empty NMeds and Cy5-labeled T3-NMeds were prepared through the nanoprecipitation procedure described above with 49 mg of PLGA and 1 mg of PLGA-Cy5. The PLGA was conjugated to Cy5 through the formation of an amide bond, as described in an already published work [26]. 

#### 2.1.4. Physico-Chemical Characterization of NMeds

NMeds were characterized in terms of their particle size, size distribution (expressed through the polydispersity index, PDI), and surface charge (expressed through the Z-potential). The NMeds were analyzed for particle size in deionized water by photon correlation spectroscopy (PCS) at 25 °C using a Zetasizer Nano ZS (Malvern Panalytical, Malvern, UK). The samples were analyzed at a NMed concentration of 0.01 mg/mL. For each analysis, three measurements were performed on three individual formulations. The Z-potential was measured with the same instrument using a combination of laser doppler velocimetry and a patented phase analysis light scattering method (M3-PALS). The same samples subjected to PCS (0.01 mg/mL) were analyzed using DTS1070 Zeta Potential cuvettes and expressed as the mean of at least three individual preparations. 

#### 2.1.5. Technological Characterization of T3-NMeds

The amount of T3 encapsulated in the NMeds was determined by extracting and quantifying the drug by UV-Vis spectrophotometric analysis (Jasco V-530, Jasco Europe, Lecco, Italy). A fixed volume (1 mL) of the NMed suspension was lyophilized without any cryoprotectant (Heto LyoLab 3000, Thermo Fisher Scientific Italia, Milano, Italy); then, 1 mL of dichloromethane (Merck Life Science, Milano, Italy) was added to the lyophilized NMeds to dissolve the PLGA and precipitate the T3, which was then centrifuged at 13,000 rpm for 10 min (Spectrafuge 24D Labnet International Inc., Edison, NJ, USA) to form a T3 pellet. The supernatant was removed, and the pellet was left to dry in a desiccator for 1 h. The dried T3 pellet was then dissolved in dimethyl sulfoxide and analyzed by UV-Vis spectrophotometry at a wavelength of 300 nm. The quantification of the T3 encapsulated was performed by interpolating the absorbance obtained using a calibration curve (T3 concentration range: 3.9–250 µg/mL). Then, the encapsulation efficiency (*EE*%) and loading content (*LC*%) were calculated: *EE* was calculated as the ratio between the amount of T3 encapsulated in NMeds compared to the amount of T3 added in formulation. The *LC* was calculated as the ratio between the amount of T3 encapsulated compared to the total weight of NMeds analyzed based on the following equations:

$$EE\%=\frac{amount\;\left(mg\right)\;of\;T3\;encapsulated}{amount\;\left(mg\right)\;of\;T3\;added\;in\;formulation}\times 100$$

$$LC\%=\frac{amount\;\left(mg\right)\;of\;T3\;encapsulated}{total\;weight\;\left(mg\right)\;of\;NMeds\;analyzed}\times 100$$

#### 2.1.6. Morphological Characterization of T3-NMeds

NMed morphology was investigated by using both atomic force microscopy (AFM) and scanning transmission electron microscopy (STEM). AFM images (Park Instruments, Sunnyvale, CA, USA) were taken at 25 °C operating in air and in non-contact mode using a commercial silicon tip-cantilever (high resolution noncontact “GOLDEN” Silicon Cantilevers NSG-11, NT-MDT, tip diameter 5–10 nm; Zelenograd, Moscow, Russia) with a stiffness of about 40 Nm^−1^ and a resonance frequency around 150 kHz. After purification, the sample was dispersed in distilled water (0.01 mg/mL) before being applied on a freshly cleaved mica disk (1 cm × 1 cm); two minutes after the deposition, the excess water was removed using blotting paper. The AFM images were obtained with a scan rate of 1 Hz. STEM analysis samples were prepared by immersing a 200-mesh Cu grid (TABB Laboratories Equipment, Berks, UK) into the NMed suspension (~0.1 mg/mL) and letting it air dry at room temperature. Images were then achieved using a Nova Nano SEM 450 (FEI Co., Hillsboro, OR, USA) (acceleration voltage 30 KV, Spot 1.5) with a scanning transmission electron microscope II detector. 

#### 2.1.7. T3 Release

The release profile of T3 was measured in synthetic ACSF buffer (artificial cerebrospinal fluid) at pH 6.8 and 7.4. The ACSF solution was prepared by dissolving 125 mM NaCl, 2 mM CaCl_2_, 3 mM KCl, 1 mM MgCl_2_, and 1.25 mM Na_2_HPO_4_ (Merck Life Sciences, Milano, Italy) in MilliQ water. Half of the solution was brought to pH 6.8 by using a diluted solution (1:10) of H3PO4 85% *w*/*w* (Merck Life Science, Milano, Italy); the other half was brought to pH 7.4 by using NaOH 0.1 N (Merck Life Science, Milano, Italy). The buffer solutions were finally filtered using Whatman nylon filters (pores 0.45 µm, diameter 47 mm; purchased from Merck Life Science) and degassed. An aliquot of the T3-NMed suspension (0.5 mL; T3 concentration 0.6 mg/mL) was placed into a dialysis tube (Spectra/Por^®^ Membranes, Spectrum Laboratories Inc., Rancho Dominguez, CA, USA) with a molecular weight cut-off of 12–14,000 Da. The tube was then placed into 150 mL of buffer and kept under gentle stirring at 37 °C. This procedure was performed in triplicate for each pH condition (6.8 and 7.4) and for each time point (1 h, 2 h, 1 day, 4 days, 1 week, and 2 weeks). At each time point, the dialysis tube was removed from the buffer, and the NMed suspension was removed from the tube and lyophilized to perform an indirect quantification of the T3 released by UV-Vis spectrophotometry (see Section 2.1.3).

### 2.2. T3-NMeds In Vitro Testing 

#### 2.2.1. T3 Concentration in NMed Suspension

T3 concentration in NMed suspension was also confirmed in buffer at pH 7.4 and pH 6.8, using an xMap commercial kit (Merck-Millipore, Milan, Italy, cat: RTHYMAG-30K) and the Luminex 200 platform. Briefly, samples were incubated overnight with micromagnetic beads conjugated with a specific primary antibody. After washing three times, a secondary antibody and Streptavidin-Phycoerythrin complex were added to the wells. The median fluorescent intensity (MFI) data obtained by Luminex^®^200^TM^ were used to calculate analyte concentrations in samples using a 5-parameter logistic curve-fitting method. Results were expressed in ng/mL.

#### 2.2.2. In Vitro Cell Experiments

We then tested the capability of NMeds to penetrate inside the cells and functionally release T3 to induce OPC differentiation. We used a cell platform based on spontaneously differentiated neural stem cells (NSCs) to analyze the NMed cellular internalization in the three neuronal lineages and the NSC-derived OPCs to test the differentiation potential of the T3-NMeds [27,28].

All protocols described herein were carried out according to the European Community Council Directives (86/609/EEC) and comply with the guidelines published in the NIH Guide for the Care and Use of Laboratory Animals.

Embryos obtained from C57BL/6J pregnant mice were decapitated with forceps, and each brain was then removed under a stereoscope and placed in a clean Petri dish. The meninges were carefully detached, the olfactory bulbs were removed, and the entire forebrain was placed in a 1.5-mL tube containing PBS and penicillin/streptomycin (pen/strep; 100 U/mL/100 µg/mL; Thermo Scientific) on ice. NSCs were isolated from the fetal forebrain as previously described [29]. The brains were collected from embryos at E13.5 and placed in a 50-mL tube containing PBS and pen/strep. The tissues were incubated with the non-enzymatic dissociation buffer (Sigma-Aldrich, St. Louis, MO, USA) for 15 min at 37 °C. The dissociated tissues were then centrifuged at 400× *g* for 5 min. 

The cell pellet was resuspended in NSC medium (DMEM/F12 GlutaMAX; 8 mmol/L HEPES; 100 U/mL/100 μg/mL pen/strep; 1 × B27; 1 × N2; 20 ng/mL bFGF; 20 ng/mL EGF; Thermo Scientific) and seeded at a density of 10 cells/µL and allowed to grow until neurospheres reached a diameter of 100–200 µm. These spheres were then mechanically dissociated and seeded again at the same density either in the NSC medium to obtain secondary neurospheres or in the OPC medium (DMEM/F12 GlutaMAX 1×; 8 mmol/L HEPES; 100 U/mL/100 μg/mL pen/strep; 1 × B27; 1 × N2; 20 ng/mL bFGF; 20 ng/mL PDGF; Thermo Scientific) to obtain oligospheres. When the spheres reached a diameter of 100–200 µm, they were dissociated into single cell suspensions and plated in 96-well plates coated with poly-D,L-ornithine (50 µg/mL)/laminin (5 µg/mL; Sigma-Aldrich) at a density of 3000 cells/cm^2^ for OPCs derived from oligospheres and 10,000 cells/cm^2^ for NSCs derived from secondary neurospheres. 

#### 2.2.3. Cell Internalization Test, Immunocytochemistry, and Confocal Imaging

NSCs derived from secondary neurospheres were seeded on glass coverslips in 24-well plates. Cells were cultivated for 7 days in NSC medium deprived of growth factors (bFGF and EGF) to induce spontaneous differentiation into the three neuronal lineages (neuronal, astroglial, and oligodendroglial lineages). Cells were incubated for 24 h with Cy5-marked NMeds at 100 μg/mL.

Cultures were fixed using 4% cold paraformaldehyde, washed twice in PBS, and stained for lineage-specific markers using immunocytochemistry. The following primary antibodies were used: mouse anti-β-III-tubulin (R&D system, Trento, Italy) 1:3000 to detect neurons; rabbit anti-GFAP (Dako) 1:1000 to detect astrocytes; rabbit anti-NG2 (chondroitin sulfate proteoglycan, neural/glial antigen 2, Millipore, Merck S.p.a., Milan, Italy) 1:350 to detect OPCs; rabbit anti-MBP (Myelin Basic Protein, Dako) 1:500 to detect mature oligodendrocytes. As secondary antibodies, donkey Alexa 488-conjugated anti-mouse IgG and donkey Alexa 488-conjugated anti-rabbit IgG, 1:500 (Invitrogen, Carlsbad, CA, USA) were used. Cells were also incubated with the nuclear dye Hoechst 33258 (1 μg/mL in PBS, 0.3% Triton-X 100) to detect the nuclei.

Confocal microscopy was used to detect and study the different cell types and the position of the NMeds. Coverslips were analyzed with a Nikon Ti-E fluorescence microscope, connected to an A1R confocal system (Nikon, Minato, Tokyo, Japan), consisting of a series of diode lasers with an output wavelength of 405 nm, an air-cooled argon-ion laser system with a 488 nm output, and a high-performance diode laser system with a 638 nm wavelength output. Images were acquired using a 40× lens with 1024 × 1024 resolution, and all z-stacks were collected in compliance with optical section separation (z-Interval) values suggested by the NIS-Elements AR 3.2 software (1 μm). To detect cell internalization, axis projection images were produced using IMARIS software (v. 9.7.2 Oxford Instruments, Abingdon-on-Thames, UK).

#### 2.2.4. Differentiation Induction Test and High Content Screening Imaging [28] 

OPCs obtained from oligospheres were seeded on 96-well plates to perform the OPC differentiation test. The standard differentiation protocol was used as a positive control condition: the OPCs were cultured in OPC medium for three days after seeding. Then, the medium was replaced with the oligodendrocyte differentiation medium (DMEM/F12 GlutaMAX 1×; 8 mmol/L HEPES; 100 U/mL/100 μg/mL Penicillin/Streptomycin; 1 × B27; 1 × N2; 50 nM T3; 10 ng/mL CNTF; 1 × N-acetyl-L-cysteine—NAC; Thermo Fisher Scientific), containing the T3 as the differentiation trigger (50 nM = 3.36 µg/mL).

For the test, cells were exposed to different concentrations of T3-NMeds, corresponding to the following T3 concentrations: 0.21, 0.42, 0.84, 1.68, 3.36, and 6.72 µg/mL.

After 12 days, cells were fixed using 4% cold paraformaldehyde, washed twice in PBS, and stained for mature oligodendrocyte-specific markers (MBP) using immunocytochemistry as described above. 

Analyses were performed using the Cell Insight™ CX5 High Content Screening platform (HCS; Thermo Fisher Scientific). Using the Compartmental Analysis BioApplication software, it was possible to identify each cell as an object based on nuclear staining. The software then detected the presence of marker-specific staining in the cell body, calculating the percentage of MBP-positive cells based on the total number of cells present in each well. The HCS system allowed for the analysis of the entire culture (10,000 to 50,000 cells/well).

### 2.3. In Vivo Study

#### 2.3.1. Animals and Surgery [30] 

Female CD-Sprague Dawley rats with 200–250 g body weight (bw) (Charles River Laboratories, Calco, Italy) were used. All animal protocols described here were carried out according to European Community Council Directives 2010/63/UE, reviewed by the Animal Welfare Body of the IRET Foundation, and approved by the Italian Ministry of Health (D.Lgs 26/2014, authorization n° 614/2019-PR). All animals were housed in an animal room on a 12-h light/12-h dark cycle at a constant temperature of 22 ± 2 °C and had food and water ad libitum. All animals were housed in pairs in plastic cages with standard bedding. One week before surgery, all animals were handled and accustomed to bladder manipulation. 

On the day of surgery, rats were pre-medicated with enrofloxacin and tramadol (4 mg/kg, s.c.), anesthetized with isoflurane (3–4%) in 2% O_2_, and then fixed in the stereotaxic table. A 4-cm-long incision was made in the skin of the back. Muscles were dissected to fully expose the T9–T11 vertebrae. The processus spinosus and lamina of T9 were removed to expose the spinal dura. A contusive lesion of the spinal cord was obtained with the Impact One impactor (Leica BioSystems, Wetzlar, Germany) using a 1.5 mm diameter tip with a force of 1 N (0.75 m/s) and 0 s of stance time; the depth of impact was 2 mm. The back muscles were sutured, and the skin incision was closed with wound clips. After surgery, rats received tramadol (4 mg/kg) for 7 days and enrofloxacin (4 mg/kg) subcutaneously (sc) for 7 days as an analgesic and to prevent infection, respectively. The control group received surgical treatment, laminectomy, and pharmacological treatments, but without SCI. The animal’s bladders were manually expressed twice a day until automatic voiding returned spontaneously. Animals were housed in single cages for the first week after surgery, then in pairs, and were monitored regularly to avoid injury. Evaluation of the rats’ wellness was performed by body weight monitoring and a clinical score [31] evaluated daily for the first two weeks, then once a week until the day of sacrifice.

To test the efficacy of the “combo” therapy designed in this study (T3-NMeds + Ibu + mNGF), we adopted the same power analysis established in a previous study [30], with locomotion as the primary endpoint. For α = 0.05, power (1-b) = 0.80, and an effect size (d) = 1.12, 14 animals per group were required (G* Power 3.1.9.2 software). A randomized surgery list was generated to assign the rats to the two cohorts, i.e., “combo” 3x therapy and vehicle. A further randomization in each cohort was performed at the sacrifice, assigning the animals to the different exploratory end-point experiments (flow cytometry, molecular biology, histology, and immunohistochemistry). We also predetermined inclusion/exclusion criteria for the lesion’s efficacy. According to the Basso–Beattie–Bresnahan (BBB) scale guidelines, in order to obtain a moderate-severity lesion in all animals, rats with a BBB score > 15 at day 7 after the lesion were excluded. The final number of animals included in each experiment is reported in the figure legends and Section 3. Intact rats were also included in selected experiments.

#### 2.3.2. T3-NMed Biodistribution and Treatments

We first tested Cy5-marked T3-NMed biodistribution in the spinal cord tissues and penetration in the different cell types following subdural injection in the T8-T9 space using confocal microscopy (instrument specifications and settings). N = 6 rats were included in this experiment. After assessing that the T3-NMeds were able to reach the site of interest, we evaluated the effectiveness of “combo” 3x therapy, where the following drugs were administered immediately after contusion:

- Subdural administration of 20 μL T3-NMeds 3.5 μg/μL (10 μL rostrally and 10 μL caudally to the lesion);
- Ibuprofen Sodium Salt (Sigma-Aldrich), through a sc implanted osmotic minipump (Alzet Model 2ML2), allowing a constant release of 120 μL/day for 21 days;
- mNGF (purified as described by [32] from adult male mouse submaxillary glands), 50 μg/kg, subdurally 20 μL (10 μL rostrally and 10 μL caudally to the lesion); then 50 μg/kg, intravenous 100 μL on postlesion days 1, 3, 5, 7, 10, 20, and 30.

#### 2.3.3. Spinal Cord Injury Functional Monitoring: BBB Score, Locomotion, and Gait Analysis

BBB Score, locomotion (open field and rotarod), and gait analysis (catwalk) tests were performed prior to the lesion and every 14 days after it [30]. The animals were observed prior to the lesion and once a week until the day of sacrifice. Each assessment was carried out by two separate operators, and the final value is based on the average of the two operators.

The BBB [33] motor function scale was used to evaluate the loss of hind limb function. This scale evaluates each parameter from joint movement, hindlimb movements, trunk position and stability, stepping, forelimb and hindlimb coordination, paw placement, and tail position, giving a score ranging from 0 to 21.

To evaluate spontaneous locomotion, the rats were tested in an open-square gray arena 46 × 46 cm (Ugo Basile, Comerio, Italy) with a brightness of 70 ± 5 Lux, where the animals were allowed to freely explore the field. Rats were placed inside individually and video recorded for 10 min. This test was performed every 14 days and analyzed using AnyMaze video tracking software v6.3 (AnyMaze, Stoelting, Wood Dale, IL, USA) to evaluate the total distance and mean speed. 

Sensory-motor coordination ability was tested using the LE 8500 RotaRod (2Biological Instruments, Varese, Italy). The rotarod test was performed by placing rats on constant rotating drums that have a textured surface to provide a suitable grip and measuring the time each animal maintained its balance on the rod. One day prior to the lesion, we trained 3 consecutive trials (1 h inter-trial interval) at a constant speed (5–10–15 rpm) for a maximum of 5 min. Post lesion tests at 28 and 56 DPL were performed at constant acceleration from 4 to 35 rpm in 5 min with a 1 h inter-trial interval. The latency to fall during the observation test was recorded.

The gait analysis was performed using the CatWalk (Noldus, Wageningen, The Netherlands) automatized system (software v.10.6). Inside an enclosed walkway, each rat ran across a glass plate three times while being recorded using a high-speed video camera that was positioned underneath the walkway. The experiment was performed in the dark while the glass walkway was illuminated, allowing the animals’ paws to reflect light as they touched the glass floor. The walkway was cleaned after each run. Footprints were analyzed, classified, and entered in the software. The Catwalk software classified paw prints with the Automatic Footprint Classification, and after that, we performed a manual correction: all traces where the limb was dragged have been excluded, and only steps with support and lifting phases have been included.

The following parameters were obtained: Regulatory Index (%), which expresses the number of normal step sequence patterns relative to the total number of paw placements; Stand (s), which expresses the duration of paw contact with the glass plate in seconds; Duty Cycles (%), which express Stand as a percentage of step cycle, and Max Contact At (%), which express the time in seconds since the start of the run that a paw makes maximum contact with the glass plate in relation to the stand of a paw. 

To enable the comparison of the runs within and between animals and reduce the variability, acceptance criteria for mean running speed and variation in running speed have been established: minimum run duration (0.50 s), maximum run duration (5.00 s), minimum number of compliant runs to acquire (3), and maximum allowed speed variation (60%). The run started when the first paws were down and ended with the last step before the animal went off-screen. 

#### 2.3.4. Tissue Sacrifice and Sampling

On the days of sacrifice (8 or 56 days post lesion, DPL), rats were anesthetized and cerebrospinal fluid (CSF) from the cistern magna was sampled. Then, the diaphragm was cut, and the pericardium was opened. Blood was collected via intracardiac sampling, and, depending on the specific purpose, the tissues of interest were collected, snap frozen, and stored at either −80 °C for gene expression studies or perfused with 4% paraformaldehyde and 14% picric acid in 0.2 M Sorensen buffer and then postfixed for 90 min for histology studies. For the spinal cord portion analyzed in flow cytometry, the tissue was isolated and immediately processed in an unaltered state.

Two different types of spinal cord sampling were applied in this study: for molecular biology analysis, western blots, and biomarker and glutamate concentration analysis, the spinal cord was dissected starting around the epicenter of the lesion and extending in portions of 10 mm in both a rostral and caudal sense. The rostral portions were identified by the letter R and an increasing number indicating the distance from the center of the injury; the same was carried out for the caudal portions identified with the letter C. For immunohistochemistry and histology analysis, a single portion of 25 mm around the core of the lesion was dissected.

### 2.4. Tissue Analysis

#### 2.4.1. Flow Cytometry

At the time of sacrifice (8 DPL), a portion extending 10 mm from the injury site was dedicated for flow cytometry analysis [30]. Briefly, animals were sacrificed and perfused with 100 mL of Dulbecco’s modified PBS (DPBS, Lonza, Basel, Switzerland) in order to remove circulating inflammatory cells. Through mechanical and enzymatic dissociation using Adult Brain Dissociation Kit solutions and GentleMACS Octo Dissociator (Miltenyi Biotec, Bergisch Gladbach, Germany), cells were isolated, centrifuged, and then resuspended at a concentration of 2 × 10^5^ cells/mL in DPBS for analysis. Cell populations were marked respectively with CD11b-PE-Vio770 (1:10), CD45-APC-Vio770 (1:10), GLAST-PE (1:10), CD32-APC (1:10), and CD86-VioBright-FITC (1:10) for the identification of macrophagic (CD11+CD45+), microglial (CD11+CD45−), lymphocytic (CD11− CD45+), astrocytic (GLAST+), and M2 lineages (CD32−/CD86−) (Miltenyi Biotec). In addition, DAPI staining solution (0.1 μg/mL) was used to visualize living cells. Immunolabeled cell count analysis was performed using MACSQuant Analyzers and FlowLogic software (Miltenyi Biotec). 

#### 2.4.2. Histology and Immunofluorescence

At sacrifice, animals were deeply anesthetized with isoflurane (3–4%) in 2% O_2_ and perfused with 4% paraformaldehyde and 14% picric acid in Sorensen Buffer 0.2 M (pH 6.9). Tissue analysis was performed both for histology and immunofluorescence by dissection of the spinal cord after at least five washes in 0.2 M Sorensen buffer containing 5% sucrose and frozen with liquid N2. A 25 mm segment was collected at the core of the lesion, 10 mm rostral and 10 mm caudal, starting at the lesion site. Longitudinal cryostat sections with a thickness of 14 μm were collected on the cryostat (Leica CM1950 Biosystems, Walldorf, Germany). Three different levels were sampled at a distance of 210 μm from each other in a dorso-ventral sense for histology and immunofluorescence studies, while for the biodistribution study, the entire spinal cord was serially sectioned. Sections were stained with Toluidine Blue and Luxol Fast Blue in order to evaluate the percentage of lesioned area and white matter loss, or processed for immunofluorescence using the following primary antibodies: anti-GFAP (Rabbit, 1:500, Dako, Santa Clara, CA, USA); anti-OX-42 (Mouse, 1:250, Serotec, Endeavour House, Kidlington, UK); anti-β-III-tubulin (Mouse, 1:250 Santa Cruz); anti-NG2 (Rabbit 1:100, Millipore, Merck S.p.a., Milan, Italy); anti-CNPase (Mouse, 1:100 Millipore, Merck S.p.a., Milan, Italy). Secondary antibodies conjugated with Rhodamine Red™-X (RRX) (1:200, Jackson Immuno Research, Cambridgeshire, UK) and Cy2 (1:200, Jackson Immuno Research, Cambridgeshire, UK) were used. Immunoreactivity analyses were performed using NIS Elements AR software (v 4.30.02, Nikon, Tokyo, Japan), and the binary area fraction was calculated for each antibody and used for analysis [30].

#### 2.4.3. Myelin Proteins Western Blot

MBP Western blot analysis was carried out according to a previously published procedure with slight modifications [11]. Proteins were isolated from the R3 portion using QIAzol Lysis Reagent (Qiagen, Hilden, Germany) after homogenization according to the manufacturer’s instructions. Briefly, the tissue was homogenized, and 180 μL of chloroform was added to each sample. Then the samples were centrifuged at 12,000× *g* for 15 min at 4 °C for separation in the three phases: an upper, colorless, aqueous phase containing RNA; a white interphase; and a lower, red organic phase containing proteins. The interphase and lower part were isolated, and ethanol was added. The DNA was sedimented by centrifugation, and the supernatant was transferred to a new test tube. Isopropanol was added to precipitate proteins, and further centrifugation was carried out. After removing the supernatant, a guanidine (0.3 M) in ethanol solution was added to the pellet and incubated for 20 min, then centrifuged twice. The pellet was resuspended in 100% ethanol, left to incubate for 20 min and centrifuged. The pellet was air-dried for 10 min, then a solution of Urea (10 M)-DTT (50 mM) was added. The solution was incubated at 95° for 3 min and then transferred to ice; it was sonicated 10 times, and a freeze/thaw cycle was performed. One last centrifugation was carried out, and the supernatant containing the proteins was moved to a new tube. For each sample, 120 µg of starting tissue and the marker protein (Precision Plus Protein Standards, Bio-Rad) were added to a solution of Laemmli/β-mercaptoethanol. The proteins were resolved in 4–20% Mini-PROTEAN TGX Stain-Free Gels (BiodRad), and the marker protein was always loaded in the external wells (lanes 1 and 10). Nitrocellulose membrane (Bio-Rad AmershamProtran 0.45 µm) was used for protein transfer. To be in line with the principles of 3Rs, thus reducing the number of samples from animals with a suffering phenotype and also reducing the amount of primary antibody, the membrane was cut after the transfer using the 37 kDa bands as a guide, and the lower portion of the membrane was processed with MBP antibody. A solution of 2.5% BSA in TBST (Tris Buffer Saline solution containing 1% Tween20) was used for 1 h of blocking. Incubation with the primary antibody (rabbit MBP DAKO, Santa Clara, CA, A0623, 1:2000) was performed overnight at 4 °C, whereas incubation with HRP-conjugated secondary antibodies (goat antiRb, BioRad-cat.1706515, 1:5000) and HRP-conjugated protein for marker visualization (Precision Protein StrepTactin HRP-conjugate, Bio-Rad, 1:10,000) was performed for 1 h at RT. Three washes with TBST were performed after incubation with the antibodies, either the primary or both the primary and secondary. Clarity Western ECL Substrate (Bio-Rad, −5 min incubation at RT in darkness) and the BioRadChemi DOC MP imaging system were used to detect the immunoreactive signal.

The Fiji (ImageJ v2.1.0) software was used to measure densitometry. The expression in percentage of the various isoforms of MBP was calculated using MPB total volume to normalize the signal.

#### 2.4.4. Glutamate Concentration

The level of free glutamate was evaluated in the R1 and C1 portions of the spinal cord tissue at 8 DPL using an enzymatic assay (Glutamate Assay Kit, Abcam, ab83389). According to the manufacturer’s instructions, the tissue was homogenized in the buffer provided in the kit, and a reaction mix with and without enzyme (blank wells) was prepared and added to the samples and standard curves. The optical density (OD) was read after a 1-h incubation at 37 °C. This kit allows a quantitative evaluation by colorimetric spectrophotometry at an OD = 450 nm. The glutamate concentration of each sample was determined using a linear standard curve interpolation (range 2–10 nmol/well). Glutamate concentrations were finally measured by a Bio-Rad DC Protein Assay (cat. 500-0116) and expressed as ng of glutamate per μg of total protein.

#### 2.4.5. Biomarkers Assay

TREM2 (triggering receptor expressed on myeloid cells 2) and NEFL (neurofilament light polypeptide) levels in the CSF were quantified at 8 DPL using commercial ELISA kits (Rat TREM2 ELISA kit, Cusabio, Cat: CSB-EL024405RA, and Rat NEFL ELISA kit, Cusabio, Cat: CSB-EL015688RA). All samples were centrifuged at 4000× *g* for 10 min at 4 °C prior to the assay and then analyzed according to the manufacturer’s indications. Briefly, undiluted samples and standard curves were added to the wells and immediately mixed with biotin antibodies. After 60 min of incubation at 37 °C, the wells were washed three times, HRP-avidin was added to each well, and the wells were incubated for 60 min at 37 °C. After incubation, the wells were washed three times, and TMB-substrate was added to each well for 30 min at 37 °C. The stop solution was added after incubation (15 min at 37 °C), and the plate was read within 15 min at 450 nm as the primary filter (subtract readings at 540 nm from the readings at 450 nm). Optical density values were interpolated on a linear standard curve using GraphPad Prism v 6.0. The TREM2 standard curve ranged from 15.6 pg/mL to 1000 pg/mL, whereas the NEFL standard curve ranged from 7.8 pg/mL to 500 pg/mL.

### 2.5. Statistical Analysis 

All data are tested for normal distribution using the Shapiro–Wilk test [34] and are expressed as mean ± SEM or Min to Max for box and whiskers. The statistical analysis and graph were generated with GraphPad Prism v8.2.1 (GraphPad Software, San Diego, CA, USA). For the parametric data, Student’s *t*-test was used for comparison of two groups, and ANOVA and post hoc tests were performed for comparison of more than two groups; for the non-parametric data, the Mann–Whitney test, Kruskal–Wallis test, and Dunn’s multiple comparison test were used. *p* < 0.05 was considered significant.

## 3. Results

### 3.1. T3-NMed Characterization

**Figure 1:**
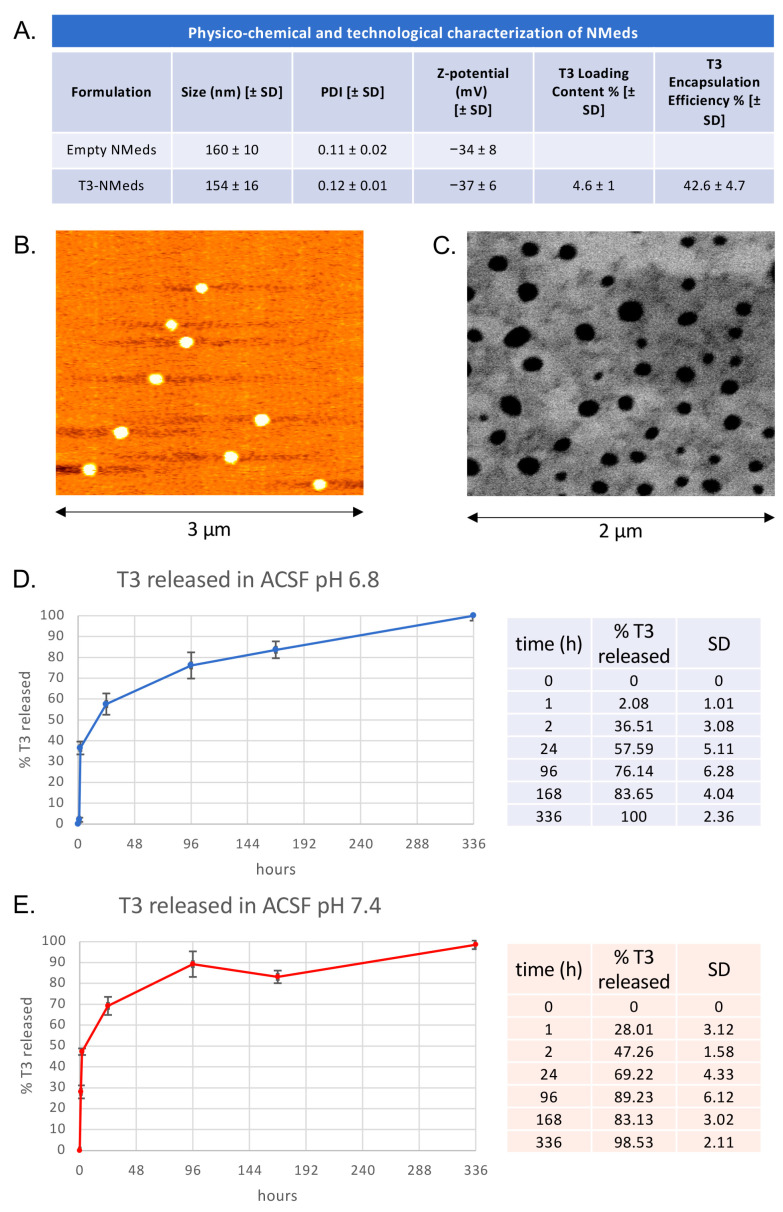
Physico-chemical, technological, and morphological characterization and T3 release profile of T3-NMeds: (**A**) Table showing the values of particle size, PDI, and Z-potential for both empty and T3-loaded NMeds and T3 encapsulation efficiency and loading content; (**B**) AFM and (**C**) STEM image of T3-NMeds; (**D**) T3 release profile of T3 released at different time points at 37 °C in ACSF buffer at pH 6.8; (**E**) T3 release profile and percentages of T3 released at different time points at 37 °C in ACSF buffer at pH 7.4. All data are represented as the mean ± standard deviation (SD), *n* = 3.

After the preparation, T3-NMeds were fully characterized in terms of their physico-chemical, technological, and morphological properties. T3-NMeds showed a particle size of 154 ± 16 nm and good homogeneity, highlighted by a PDI of 0.12 ± 0.01. The Z-potential was found to be negative, around −40 mV. Furthermore, it can be noticed that the encapsulation of the drug had no negative impact on the physico-chemical properties of NMeds, resulting in T3-NMeds with low particle size and PDI, comparable to the empty NMeds. Additionally, the Z-potential did not change after the addition of T3 (Figure 1A). Regarding the drug loading, we observed that the EE% and LC% of T3 were, respectively, around 40–45% and 5% (Figure 1A). Morphological investigation by AFM and STEM images confirmed the good homogeneity of the NMed population and showed the spherical shape of T3-NMeds (Figure 1B,C). The release of T3 from the NMeds was investigated at two different pHs (6.8, to mimic a slightly acid pH that characterizes a tissue where an inflammatory process is underway; and 7.4, that is the physiological pH) in ACSF buffer at 37 °C over 14 days. At both pH 6.8 and pH 7.4, a burst release of T3 was observed in the first 2 h, reaching a release of 36 and 47%, respectively. A further 20% was released between 2 and 24 h, followed by a more constant release that led to 100% of T3 being liberated at 14 days (Figure 1D,E).

### 3.2. In Vitro T3 Release and Cell Uptake

**Figure 2:**
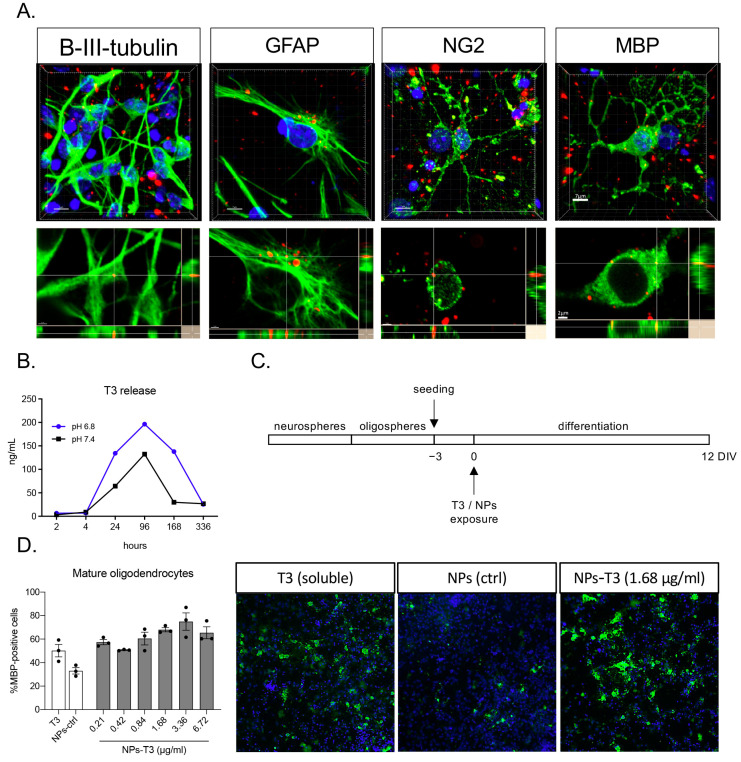
NMeds internalization and T3 release in vitro: (**A**) Representative images of NMeds (red) internalized in all the different neural lineages derived from neural stem cell differentiation. Cell lineages were identified by specific markers (green): beta-III-tubulin for neurons, GFAP for astrocytes, NG2 for oligodendrocyte precursor cells, and MBP for mature oligodendrocytes. Images are z-stacks acquired by confocal microscopy and elaborated by IMARIS software. For each lineage, the maximum intensity projection-based 3D image and the lateral projections of the axis are represented, showing the red spots corresponding to the NMeds inside the cell cytoplasm. Bars: 7 μm for z-stacks and 2 μm for lateral projections; (**B**) T3 release with the xMap kit at pH 6.8 and pH 7.4; (**C**) protocol used for the in vitro differentiation experiment; (**D**) differentiation of the neural stem cell-derived oligodendrocytes expressed as percentage of MBP-positive cells on the total cell number identified by the Hoechst nuclear staining after treatment with the standard differentiation protocol (soluble T3, 50 nM, 3.36 µg/mL), control NMeds, or different concentrations of T3-NMeds. Representative images acquired by cell-based high-content screening. The brightness and contrast of the pictures were adjusted to increase the quality of the image visualization without affecting the qualitative and quantitative analyses that were performed on the raw images. Abbreviations: GFAP—glial fibrillary acidic protein; MBP—myelin basic protein; NG2—neural/glial antigen 2; NPs—nanoparticles; T3—triiodothyronine.

The intracellular distribution of clusters of Cy5-labeled T3-NMeds was confirmed in vitro in GFAP-positive astrocytes and differentiating OPCs (NG2 and MBP-positive), which were derived from neural stem cells (Figure 2A). The 3D projection images obtained from the confocal microscopy z-stack acquisition mode confirmed NMeds internalization (Figure 2A, lower panels).

Next, the in vitro release of T3 was investigated by an xMap commercial kit. T3 release was measured in buffer at both pH 6.8 and 7.4. A more sustained release was observed in the more acidic buffer, while in both conditions, a release peak was observed after 96 h (Figure 2B). The same model was used for the efficacy test in the OPC differentiation assay coupled with the cell-based HCS, proving that the T3 released by the NMeds induces OPC differentiation in mature oligodendrocytes (Figure 2C,D). This is a well-established in vitro model that is highly sensitive to the presence of T3 in the culture medium. It has been proven, in fact, that the NSC-derived OPC in vitro retains the need for T3 stimulus to properly differentiate and mature [29], mimicking the in vivo differentiation mechanisms. The system can be modulated and adapted to different methodological needs, mimicking physiological and pathological conditions [27,29]. Moreover, it can be combined with the HCS technology, which automatically detects and analyzes all the cells in the culture, removing the bias of acquiring random representative fields [28].

**Figure 3:**
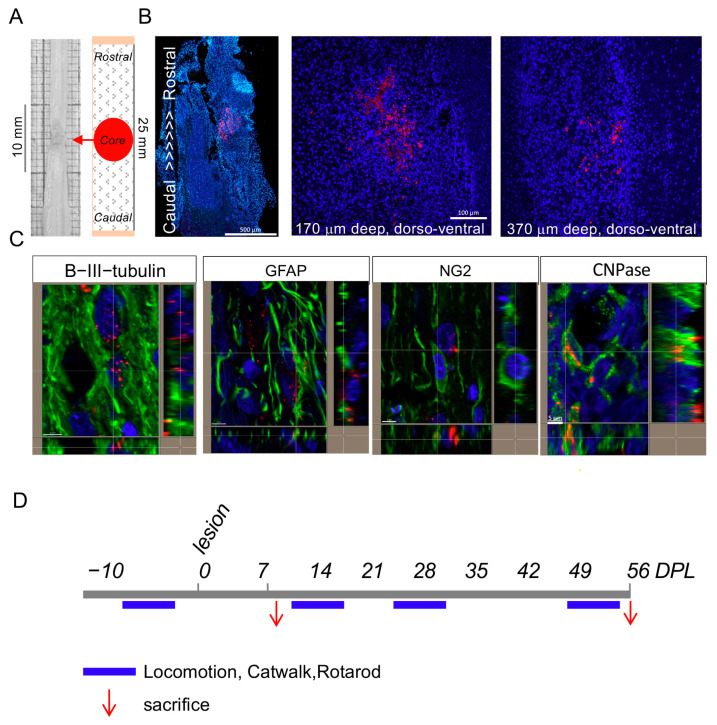
Biodistribution of NMeds in the rat model of SCI: (**A**) The sampling schema for tissue processing is shown; (**B**) biodistribution of the Cy5-marked T3-NMeds in the spinal cord tissue at different dorso-ventral levels; (**C**) representative confocal microscopy images of T3-loaded NMeds internalization in the target cells, i.e., neurons (βIII-tubulin), astrocytes (Gfap), oligodendrocyte precursor cells (NG2), and mature oligodendrocytes (CNPase). Bars: 500 μm for image montage, 100 µm for single images, and 5 µm for lateral projections; (**D**) experimental schedule pointing out the timing for tissue and biological fluid analysis (red arrows) and locomotion testing (blue bars). Abbreviations: CNPase—2′,3′-cyclic-nucleotide 3′-phosphodiesterase; DPL—days post lesion; GFAP—glial fibrillary acidic protein; NG2—neural/glial antigen 2.

The biodistribution of the Cy5-marked T3-NMeds following subdural injection in SCI rats was investigated in order to determine if the NMeds could diffuse from the injury site and enter the cells. In Figure 3A,B, the presence of NMeds (red dots) inside the tissue is shown. Moreover, T3-loaded NMeds were uptaken by the target cells, i.e., neurons, astrocytes, OPCs, and mature oligodendrocytes (Figure 3C), while NMeds were not detectable in the blood or CSF. The biodistribution was performed at both short- (8 DPL) and long-term (56 DPL) (Figure 3D).

### 3.3. Effect of 3× “Combo” Therapy on Neuroinflammation

**Figure 4:**
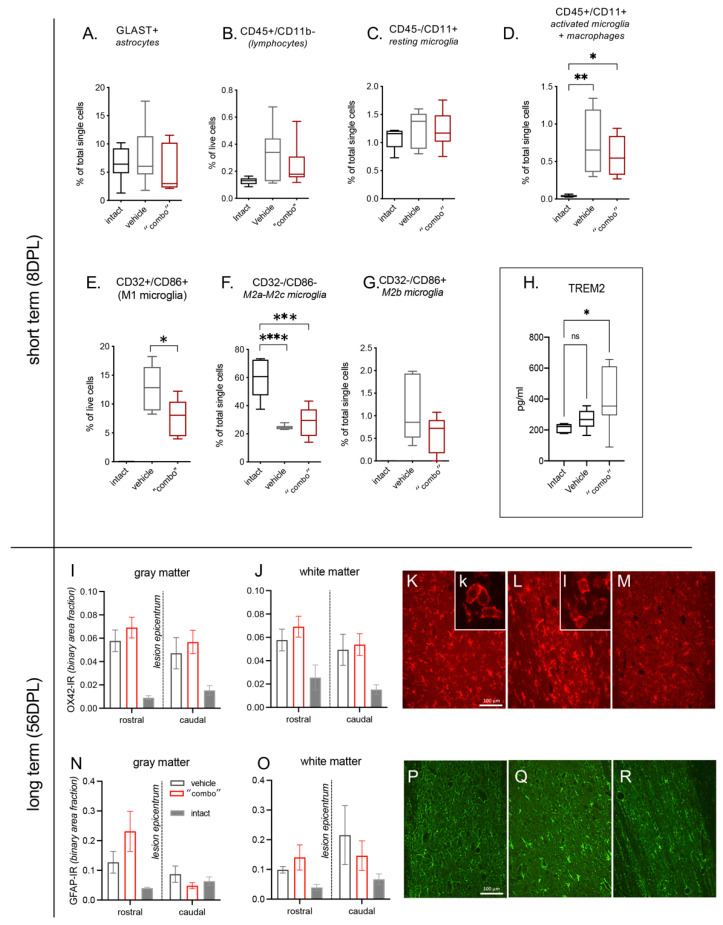
Effect of 3× “combo” (T3-loaded NMeds-mNGF-Ibu) on inflammatory markers at short and long term. The graphs show the % of positive cells for a specific marker on total counted cells by flow cytometry analysis of the spinal cord tissue at 8 DPL: (**A**) GLAST+ for astrocytes; (**B**) CD45+/CD11b− for lymphocytes; (**C**) CD45−/CD11b+ for resting microglia; (**D**) CD45+/CD11b+ for microglia activated and macrophages; (**E**) CD32+/CD86+ for M1 microglia; (**F**) CD32−/CD86− for M2a-M2c microglia; (**G**) CD32−/CD86+ for M2b microglia; (**H**) TREM2 levels in the CSF; (**I**–**M**) relative binary area fraction of OX42-IR referring to microglia in the gray (**I**) and white (**J**) matter, analyzed rostrally and caudally to the lesion epicentrum. Representative micrographs from vehicle (**K**), “combo” (**L**), and intact (**M**) groups are shown, where the inserts illustrate the prevalent cell morphology; (**N**–**R**) relative binary area fraction of GFAP-IR referring to astrocytes in the gray (**N**) and white (**O**) matter, analyzed rostrally and caudally to the lesion epicentrum. Representative micrographs from vehicle (**P**), 3× “combo” (**Q**), and intact (**R**) groups are shown. Bars: 100 µm. Data are presented as boxplot (cytofluorimetric analysis, N = 7 rats/group) or mean + SEM (immunohistochemistry, N = 8 rats/group). Statistical analysis: Kruskal–Wallis test; Student’s *t*-test; *p <* 0.05 was considered significant (* *p* < 0.05; ** *p* < 0.01; *** *p* < 0.001 **** *p* < 0.0001). The brightness and contrast of the pictures were adjusted to increase the quality of the image visualization without affecting the qualitative and quantitative analyses that were performed on the raw images. Abbreviations. DPL—day post lesion; TREM2—triggering receptor expressed on myeloid cells 2.

To verify whether the 3× “combo” therapy (T3 NMeds, Ibu, and mNGF) could regulate the early inflammatory cellular responses in the spinal cord, we analyzed cells expressing inflammation-related membrane markers by flow cytometry analysis at 8 DPL (N = 7 rats/group) (Figure 4A–G).

First, we analyzed astrocytes (GLAST+ cells; Figure 4A), total lymphocytes (CD45+/CD11b−; Figure 4B), and resting microglia (CD45−/CD11+; Figure 4C), and no significant differences were detected. However, when we went deeper in the analysis of the microglia subpopulations, we described that the activated microglia + macrophages increased due to the lesion without any effects of the treatment (CD45+/CD11+ One-way ANOVA, F(2,16) = 10.83; *p* = 0.0011, Tukey’s post-test, intact vs. vehicle, *p* = 0.0012; intact vs. 3× combo, *p* = 0.0108; Figure 4D).

The cell population corresponding to type M1 microglia is not represented in intact animals (all analyzed samples presented “0” as a value of quantification, thus this group was excluded from the stastical analysis). However, M1 cells show a significant decrease in combo treatment animals compared to vehicle (CD32+/CD86+ Student’s *t*-test; *p* < 0.05; Figure 4E).

The M2a-M2c microglia population is highly represented in the intact group while decreasing in both vehicle and “combo”-treatment animals (CD32−/CD86− One-way ANOVA, F(2,18) = 22.79; *p* < 0.0001; Tukey’s post-test, intact vs. vehicle, *p* < 0.0001; intact vs. 3× combo, *p* = 0.0001; Figure 4F). The microglia subpopulation M2b (CD32−/CD86+; Figure 4G) is not represented in intact animals (all analyzed samples presented “0” as a value of quantification, thus this group was excluded from the stastical analysis). Even if the lesion generated a slight increase (almost 2%), and the analyzed samples derived from “combo”-treated animals show a decrease (around 1%), this difference is not statistically significant.

Taking all these data together, it is possible to conclude that type M1 microglia were strongly reduced, although not suppressed, by the “combo” treatment. Levels of the microglia-associated marker TREM2 in the CSF at 8 DPL also showed a significant increase in treated rats (Figure 4H).

We then explored the residual neuroinflammation in the spinal cord tissue rostrally and caudally to the lesion epicentrum at long term (56 DPL) using OX42-IR as a microglia marker (Figure 4I–M) and GFAP-IR for astrogliosis (Figure 4N–R). In both vehicle and 3× “combo” treated animals, we observed an increase in immunostaining compared to non-injured rats, and no substantial differences were observed in the microglia morphological phenotype between vehicle and 3× “combo” treated rats.

### 3.4. Effect of the 3× “Combo” Therapy on Demyelination/Remyelination

**Figure 5:**
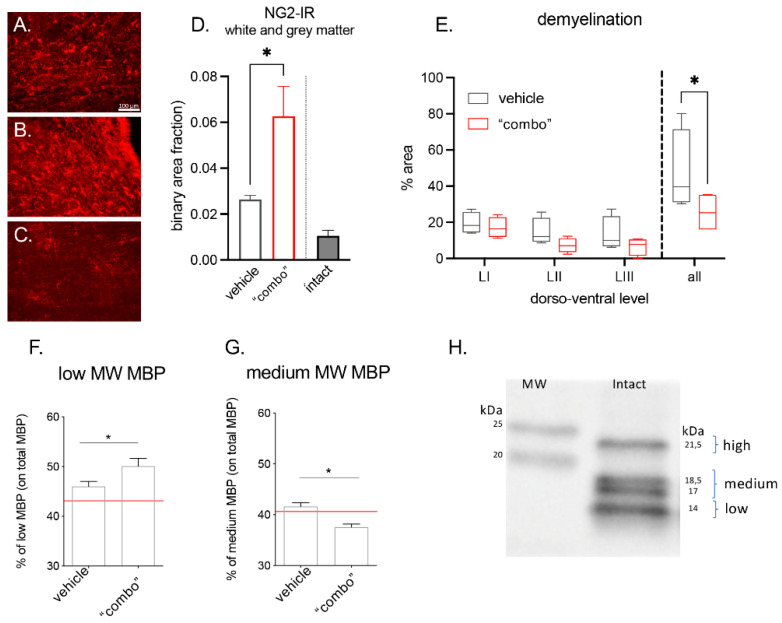
Effect of the 3× “combo” therapy on remyelination markers at long term: (**A**–**C**) Representative micrographs of NG2-IR from (**A**) vehicle, (**B**) “combo”, and (**C**) intact groups are shown. Bars: 100 µm; (**D**) relative binary area fraction of NG2-IR referring to OPCs in grey and white matter, close to the lesion area in the spinal cord; (**E**) demyelinated area evaluated in Luxol Fast blue stained sections: relative demyelinated area at three different dorsoventral levels (LI, LII, LIII), and the overall ratio (all), in vehicle and “combo”-treated rats; (**F**–**H**) graphs showing the % of low and medium isoforms on the total MBP in vehicle and “combo”. The red line represents the intact rat value; (**H**) representative Western Blot showing the different MBP isoforms. Data are presented as the mean + SEM (immunohistochemistry N = 8 rats/group of treatment, N = 6 intact; WB N = 7 rats/group) or as boxplot (histochemical analysis N = 5 rats/group). Statistical analysis: Student’s *t*-test; * *p* < 0.05; *p <* 0.05 was considered significant. Brightness and contrast of the pictures were adjusted to increase the quality of the image visualization without affecting the qualitative and quantitative analysis which were performed on raw images. Abbreviations: MBP—myelin basic protein; MW—molecular weight; NG2—neural/glial anti-gen 2.

Myelin protection and/or repair obtained by the 3× “combo” was analyzed long term after lesion by immunohistochemistry of the OPCs using the markers NG2 (Figure 5A–D), Luxol Fast blue histochemical staining (Figure 5E), and MBP western blot (Figure 5F–H).

We observed that the NG2-IR signal strongly increased in treated rats, given that the cells were more numerous and hypertrophic. Demyelination area was measured in 25 mm rostro-caudal longitudinal sections having the lesion as the epicentrum, with measurements at three different dorsoventral levels (LI being the most dorsal and LIII the most ventral). We observed that the demyelinated area was gradually reduced and the overall value was significantly lower in the 3× “combo” group compared to vehicle-treated rats.

The myelin basic protein consists of four different isoforms with the following molecular weights: 21.5 kDa, 18.5 kDa, 17 kDa, and 14 kDa [35,36,37]. We analyzed the relative abundance of the 21.5 kDa (named high isoform), 18.5 kDa, and 17 kDa quantified together (named medium isoform), and the 14 kDa isoform (named low isoform) by western blot analysis. In 3× “combo”-treated rats, the 14 kDa increased (Figure 5F), whereas the 18.5–17 kDa significantly decreased.

### 3.5. Effect of the 3× “Combo” Therapy on Neuroprotection

**Figure 6:**
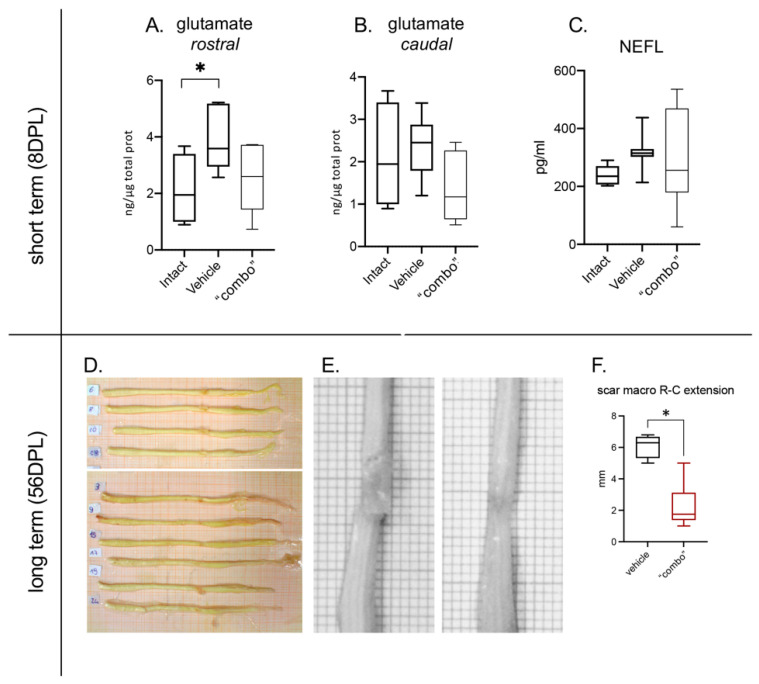
Effect of the 3× “combo” therapy on neurodegeneration markers, short- and long-term. (**A**,**B**) Tissue levels of glutamate in the rostral and caudal spinal cord segments respective to the lesion epicentrum at 8 DPL; (**C**) NEFL in the CSF at 8 DPL; (**D**,**E**) photographs of the entire spinal cord of vehicle ((**D**), upper; (**E**), left) and treated ((**D**), lower; (**E**), right) treated rats. (**F**) Rostrocaudal extension of the scar in vehicle and “combo”-treated rats. Data are presented as boxplots (glutamate N = 7 rats/group; NFL N = 7 rats/group; scar N = 5 rats/group). Statistical analysis: A: One-way ANOVA and F: Mann–Whitney test; * *p* < 0.05; *p <* 0.05 was considered significant. Abbreviations: DPL—day post lesion; NEFL—neurofilament.

To evaluate neuroprotection, we first dosed glutamate levels in the spinal cord segments R1 and C1 to the lesion at short term after the lesion. We observed that the 3× “combo” therapy tended to counteract the increase of lesion-induced glutamate, particularly in the rostral segment; however, the effect was not significant due to the high variability of the data (Figure 6A,B). Similarly, NEFL variations in the CSF at 8 DPL among the groups were not significant (Figure 6C).

We then evaluated the scar extension at 56 DPL, representing lesion stabilization, by macroscopical visual analysis after spinal cord sampling (Figure 6D,E), from both vehicle and treated animals. The analysis showed a significant reduction in the area of the scar in treated rats compared to vehicle-injected groups (Figure 6F).

### 3.6. Effect of the 3× “Combo” Therapy on the Functional Outcome

**Figure 7:**
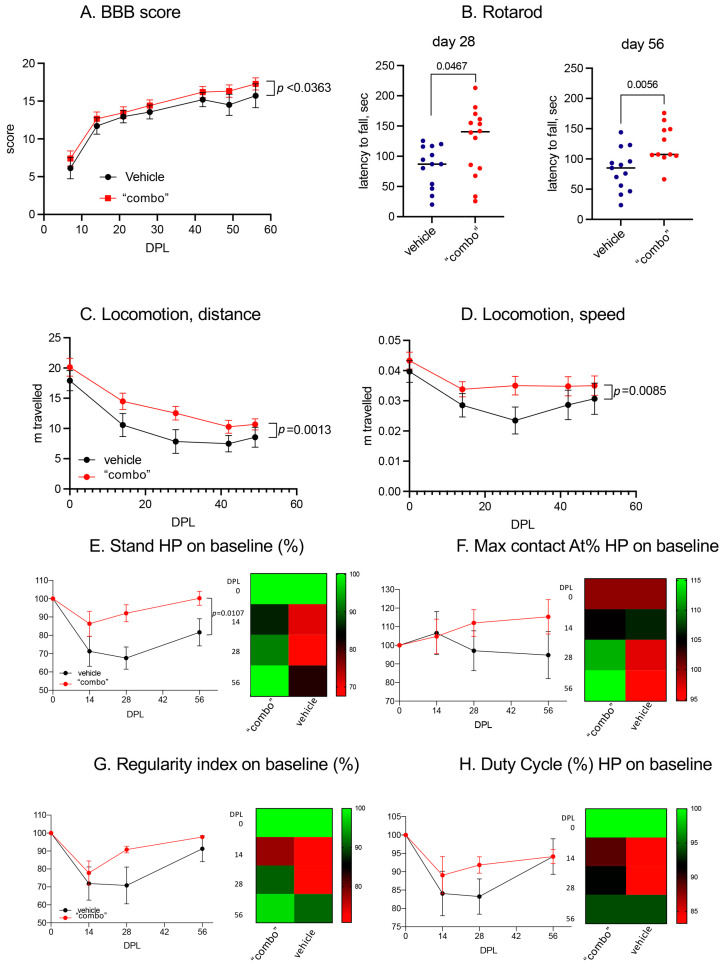
Effect of the 3× “combo” therapy on the functional outcome long term. (**A**) BBB score over the course of the experiment in rats treated with vehicle and “combo”; (**B**) sensory motor integration evaluated by the Rotarod test, evaluated at 28 and 56 DPL; (**C**,**D**) spontaneous locomotion expressed as distance traveled and mean speed; (**E**–**H**) Gait analysis showing the kinetic and coordination parameters at 14, 28, and 56 DPL, including stand value, max contact AT (%), regularity index (%), and duty cycles (%). Data are expressed as mean + SEM, N = 13 rats/group; statistical analysis: two-way ANOVA; *p <* 0.05 was considered significant. Abbreviations: BBB—Basso–Beattie–Bresnahan; DPL—day post lesion; HP—hind paws.

We finally monitored the functional effect of the therapy by evaluating different locomotion parameters (Figure 7).

Using the conventional BBB index through a subjective numeric scale ranging from 0 (complete paralysis) to 21 (normal locomotion), which explores the rats’ attitudes toward locomotion (Figure 7A), we observed a slight but significant improvement over the entire observational period. This raw observation was confirmed by the spontaneous locomotion analysis, in which we observed that both the distance traveled and the mean speed improved in the 3× “combo” treated rats compared to those treated with only the vehicle (Figure 7C,D).

We then moved to more challenging locomotor tests, such as the RotaRod, which explored sensory motor integration in a forced, dynamic condition. In this test, the 3× “combo”-treated rats performed much better than the vehicle-treated rats at both explored post-lesion times (Figure 7B). The gait was also analyzed by CatWalk, where the 3× “combo” showed a positive effect on all the parameters analyzed, with a more rapid return to baseline values (recorded before the injury). At 14 DPL, no differences were noted in the two groups for any of the parameters analyzed, while at 28 DPL, the positive effect of the treatment became evident, with treated animals recovering their baseline values at 56 DPL. The stand value of the hind limbs after injury (Figure 7E) was significantly reduced (two-way ANOVA, F(1.35) = 7.2; *p* = 0.0107) in the untreated group compared to the treated animals. The regularity index (RI) indicated the degree to which an animal used their four paws, considering the order of paw placements. Duty Cycle (DC) indicated the percentage of time spent in support during walking. In both RI and DC, overall values were positively affected by treatment but did not reach statistical significance due to the large variability in the untreated animal values (Figure 7G,H). One the Max Contact AT% parameter, the treated animals showed an increase compared to both the baseline and the untreated animals starting from 28 DPL (Figure 7F). The Max Contact can be considered the point when the braking phase turns into the propulsion phase. The trend between the two groups suggested that the treated animals started using the hind limbs again during the gait, not only for support but also for propulsion (28 DPL).

## 4. Discussion

In this study, we tested a multitarget approach to limit secondary degeneration onset and progression in experimental SCI. We used a combination of marketed drugs and a novel administration solution, which were delivered immediately after the experimental injury. T3-loaded NMeds were locally injected at the lesion site with the aim of favoring the differentiation of OPCs and myelin repair; the systemic administration of ibuprofen was aimed at reducing the detrimental effect of inflammation; and local and systemic NGF was administered to favor neuroprotection and modulate inflammation. This 3× “combo” therapy was demonstrated to reduce lesion-induced microglia type 1 activation while also increasing TREM2 concentration in the CSF at short term (8DPL), favoring myelin protection/repair, and recovering locomotor functionality at long term (56 DPL).

The primary target of this drug combination was white matter protection and repair. White matter degeneration and progressive demyelination are hallmarks of SCI, altering neuronal function and long-distance input transmission. The disruption of the molecular and cellular interactions among the axon, associated neuronal cell body, and myelinating oligodendrocytes leads to progressive and long-lasting axonal degeneration, also in distant sites from the soma [38]. Thus, myelin repair is a major therapeutic target in SCI to counteract functional deficits, as well as in other conditions characterized by severe white matter damage [39]. Myelin repair in the adult CNS is linked to the recruitment and activation of resident OPCs that can differentiate and replace lost oligodendrocytes [40]. However, the differentiation of OPCs frequently fails in the case of inflammatory-demyelinating diseases due to the complex microenvironmental alterations [41,42]. Based on the well-established role of TH in inducing OPC cell cycle exit and terminal differentiation [43,44], we developed a rationale for the use of THs by systemic supplementation to favor myelin repair in different pathological conditions characterized by white matter damage, confirming the effectiveness of THs as remyelinating agents [13,14,45,46,47,48]. Notwithstanding their beneficial effects, the potential thyrotoxicity induced by systemic administration of THs hampers the translational potential of this approach. Thus, we developed an innovative T3-delivery solution designed to release appropriate concentrations of the drug at the site of the lesion for at least 14 days. We optimized PLGA-based NMeds loaded with T3, which were fully characterized in terms of physico-chemical, morphological, and technological properties. Results showed that we obtained NMeds with a particle size near to 150 nm and a low PDI (<0.15), indicating a narrow size distribution and good homogeneity [49,50]. These properties, associated with the spherical shape of NMeds confirmed by the AFM and STEM investigations and with a strongly negative Z-potential [51], suggest that these NMeds are suitable for both storage and in vivo administration. T3 was successfully loaded into NMeds with over 45% encapsulation efficiency and an LC% of almost 5%, indicating that 5% of the NMed matrix is composed of T3. We explored the T3 release profile from NMeds in ACSF buffer at two different pHs (6.8, to mimic a slightly acidic pH that characterizes an inflamed tissue, and the physiological 7.4) at 37 °C. As expected for polymer-based NMeds [52], we noticed a burst release of the drug at both conditions tested during the first 24 h of incubation, leading to the release of about 60% and 70% of T3 at pH 6.8 and 7.4, respectively. Nevertheless, the developed NMeds could release the drug over a 14-day period, with a more controlled and constant release after the first day.

We first explored nanoparticle uptake and biodistribution using Cy5-labeled T3-NMed. Cy5-T3-NMeds were able to penetrate different cell types, including OPCs, in vitro and in vivo. When tested in vitro, T3-NMed induced OPC differentiation to the same extent as soluble T3 (3.36 µg/mL) in a dose range from 0.21 to 6.72 µg/mL. When administered in vivo, Cy5-T3-NMed could diffuse from the subdural injection side to the nervous tissue and could be detected rostrocaudally and dorsoventrally for 1450 and 770 μm, respectively. Cy5-NMeds were observed inside neurons, astrocytes, and oligodendrocytes. The local delivery of T3 in the spinal cord has also been proposed through a hydrogel-based drug delivery system, allowing a prolonged (over 70 days) release at doses of 1.51 and 0.88 µg/kg/d in the first 2 days after implantation, thus around 0.57 µg/kg/d [53]; however, we have furthered the knowledge regarding this system since in the previous works functional data were not studied.

The second target of the “combo” therapy was inflammation. Inflammation after SCI is driven by a diverse set of cells and signaling molecules [50], leading to several detrimental effects such as tissue damage, increased vascular permeability, neuron, OPC, and OL apoptosis and cytotoxicity, lipid peroxidation, mitochondrial damage, etc. Moreover, the activity of THs on lesioned spinal cord tissue is severely hampered by inflammation, leading to tissue hypothyroidism and a condition known as “non thyroidal illness syndrome” [15,54]. Inflammation leads to an increase in the expression of the thyroid hormone-inactivating enzyme deiodinase 3 and a decrease in the expression of thyroid hormone receptors (THRs) [51], and numerous in vitro and in vivo studies have indicated that inflammatory cytokines impair OPC differentiation and the expression of myelin-related genes [29,55,56,57,58]. Finally, inflammatory cells are a major source of glutamate, which promotes excitotoxicity [59]. Regarding the choice of the anti-inflammatory drug, ibuprofen was selected, as corticosteroids have been abandoned due to inconsistency in functional improvement and gastrointestinal bleeding risk [7], and glucocorticoids are associated with decreased white matter integrity [60]. On the contrary, NSAIDs have attracted great interest for the treatment of neurological diseases [61]. In the context of SCI, ibuprofen, whether systemically or locally delivered, was demonstrated to decrease edema and inflammation, promote axon germination and nerve fiber regeneration after thoracic spinal cord injury, reduce the formation of syringomyelia, and protect white matter, thereby improving locomotor recovery [30,62,63].

NGF was also included in the 3× “combo” therapy thanks to its multiple effects in modulating inflammation and immune reactions [64] also regulating the lesion-induced inflammatory responses in SCI [65]. Moreover, NGF is neuroprotective for several neural populations [64], including sensory spinal tracts such as the *fasciculus gracilis* and *fasciculus cuneatus*, and the ventral and lateral spinothalamic tracts [66]. Finally, several studies underline that therapies able to increase endogenous NGF (e.g., via genetically modified cells or tissue engineering scaffolds [67] or by local NGF administration [68,69,70]) can improve the functional outcome in SCI [10,67].

The tissue analysis shortly after the injury indicated that 3× “combo” was effective in modifying key players in secondary degeneration induction and progression. For example, the lesion-induced increase of glutamate in tissues was prevented in treated rats, reducing the effects of glutamate excess such as the triggering of cell death associated with secondary degeneration, involving the NMDA receptor in neurons [71], AMPARs in oligodendrocytes [59], and Ca^++^ overloading as a common mechanism [72]. This role of the 3× “combo” in mitigating secondary degeneration is reinforced by results on the functional outcome. After the initial traumatic damage, 3× “combo”-treated rats show a minor deficit compared to animals treated with vehicle, and a progressive improvement is evident at 28 DPL, which is maintained at 56 DPL.

Another remarkable effect we observed was type M1 macrophage/microglia reduction at the lesion site, as described by tissue flow cytometry immunophenotyping. The M1 macrophage/microglia phenotype induces inflammation and worsens tissue damage by producing pro-inflammatory cytokines [73], while the transition from the M1 to the M2 phenotype of macrophages/microglia supports the regression of inflammation and tissue repair [74]. Notably, microglia-associated triggering receptor expressed on myeloid 2 (TREM2) strongly increased in the CSF of treated rats. TREM2 is an immunoglobulin receptor that acts as a regulator in the inflammatory response and phagocytosis, and its upregulation characterizes microglia that protect neurons from damage [75].

More recently, a key role for TREM2-microglia has also been proposed for OPC protection and remyelination in SCI [76]. In fact, demyelination is persistent in TREM2 knockout mice [77], while TREM2 activation on microglia increased the density of OPC in areas of demyelination as well as the formation of mature oligodendrocytes, thus enhancing remyelination and axonal integrity [78]. In fact, in 3× “combo”-treated rats, we observed a higher tissue density of NG2-positive OPCs at long term after injury, indicating that resident OPCs start to proliferate as expected after injury [79], survive in the tissue microenvironment made less hostile also by the anti-inflammatory drug, and differentiate as supported by T3-nanomedicine, as indicated by increased biochemical indices of remyelination (MBP level) and reduced demyelination at long term after SCI. At the end, the sum of these molecular and cellular effects could also play a role in reducing scar formation and improving functional outcomes.

## 5. Conclusions

In this PoC study, we demonstrated that multiple treatments designed to target more than one pathogenic mechanism inducing secondary degeneration can be effective in improving the functional and anatomical outcome in experimental contusion SCI models. T3-loaded NMeds locally injected at the lesion site, associated with the systemic administration of ibuprofen, and local and systemic NGF are effective on several recognized markers of SCI progression, finally resulting in an improved functional outcome. Moreover, we also demonstrated that NMeds can successfully be used to obtain a more controlled local drug delivery that would normally result in more widespread toxicity via traditional systemic administration routes.

Results obtained from this drug combination, and in particular the effectiveness of NMeds-based DDS, foster other “combo” schemes based on the repurposing principle, i.e., including anti-glutamatergic drugs available for other diseases that are burdened by severe side effects unacceptable for SCI patients.

## Data Availability

Raw data are available from the corresponding authors upon reasonable request.
